# Supplementary material for: Inhibitory Effect of Osthole from Cnidium monnieri on Tobacco Mosaic Virus (TMV) Infection in Nicotiana glutinosa
Source: Molecules. 2019 Dec 24;25(1):65. doi: 10.3390/molecules25010065 (PMC6982833; doi:10.3390/molecules25010065)
Supplement: Supplementary file 1 [file molecules-25-00065-s001.pdf]

molecules

MDPI

Article

Inhibitory Effect of Osthole from *Cnidium monnieri* on Tobacco Mosaic Virus (TMV) Infection in *Nicotiana glutinosa*

Ya-Han Chen [1,2], Dong-Sheng Guo [2], Mei-Huan Lu [2,3], Jian-Ying Yue [1], Yan Liu [4], Chun-Ming Shang [4], De-Rong An [2,*] and Ming-Min Zhao [1,*]

[1] College of Horticulture and Plant Protection, Inner Mongolia Agricultural University, Hohhot 010019, China; yhchen1018@nwafu.edu.cn (Y.-H.C.); yuejianying2018@163.com (J.-Y.Y.)

[2] College of Plant Protection and State Key Laboratory of Crop Stress Biology for Arid Areas, Northwest A&F University, Yangling 712100, China; gds1995908@163.com (D.-S.G.); lu_meihuan@sina.com (M.-H.L.)

[3] Microbial Resources of Research Center, Microbiology Institute of Shaanxi, Xi'an 710043, China

[4] Academy of Agriculture science in Baotou, Baotou 014010, China; liuyanww@126.com (Y.L.); chunmingsh@163.com (C.-M.S.)

* Correspondence: anderong323@163.com (D.-R.A.); Mingminzh@163.com (M.-M.Z.); Tel.: +86-158-2909-7529 (D.-R.A.); +86-157-7136-0659 (M.-M.Z.); Fax: +86-029-8708-2710 (D.-R.A.); +86-0471-638-5801 (M.-M.Z.)

Supplementary Materials

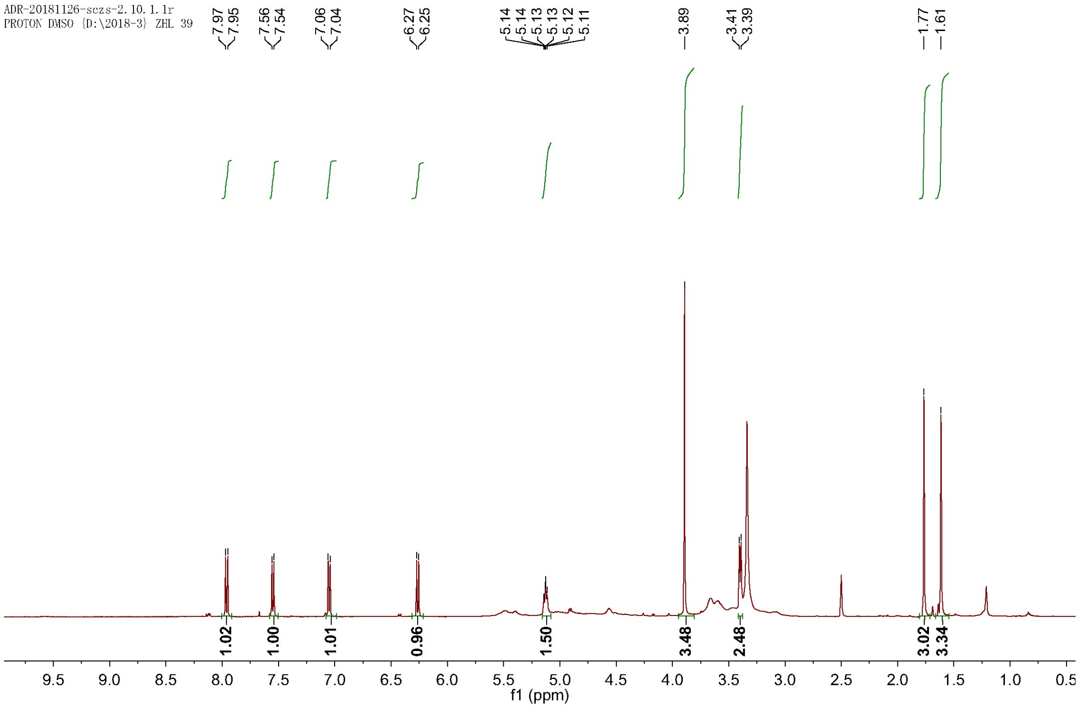

Figure S1. H-NMR of osthole.

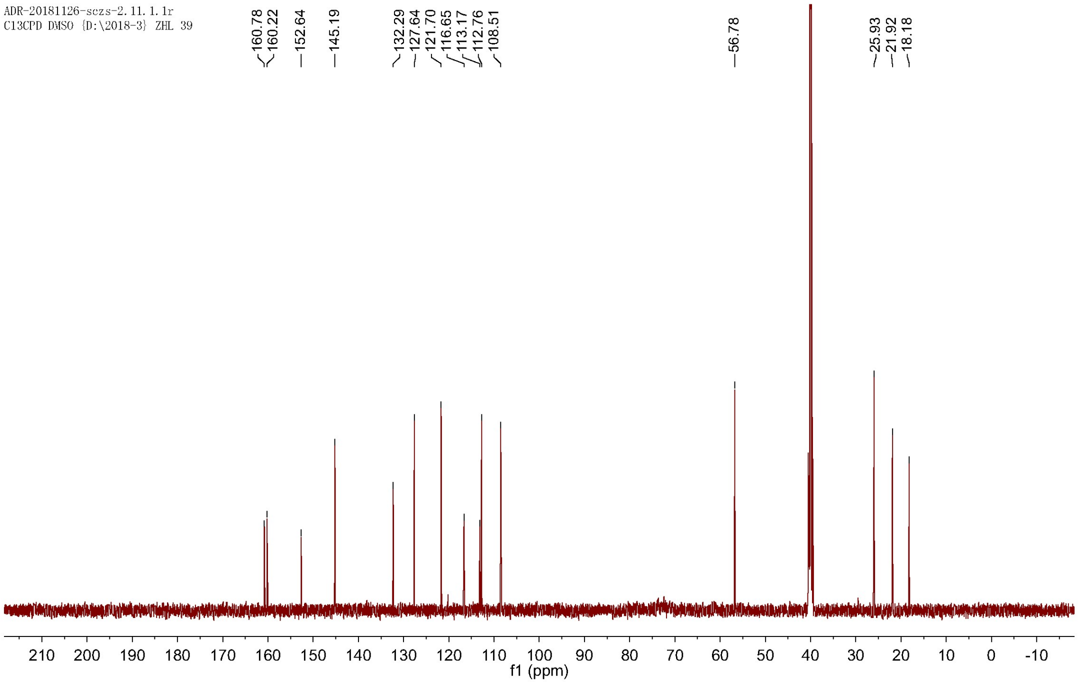

Figure S2. C-NMR of osthole.
